# Successful use of recombinant activated factor VII (eptacog alfa, Novoseven®) in a refractory bleeding during pediatric cardiac surgery: a case report

**DOI:** 10.1186/s40981-015-0012-7

**Published:** 2015-11-30

**Authors:** Mariko Ishihara, Yoshikazu Miyamoto, Akihiro Taniguchi, Keiko Kinouchi

**Affiliations:** 1 Department of Anesthesiology, Shiga University of Medical Science, Seta Tsukinowa-cho, Otsu, Shiga Japan; 2 Department of Anesthesiology, Osaka Medical Center and Research Institute for Maternal and Child Health, 840 Murodocho, Izumi, Osaka Japan

**Keywords:** Pediatric cardiac surgery, Refractory bleeding, rFVIIa, Acquired hemophilia A

## Abstract

A 2-year-and-7-month-old boy underwent an emergent reconstruction surgery of the right ventricle-pulmonary artery (RV-PA) conduit. Although he was successfully weaned from cardiopulmonary bypass (CPB) after reconstruction of the RV-PA conduit, the bleeding continued despite the massive transfusion of red blood cell (RBC), fresh frozen plasma (FFP), and platelets. Because of persistent bleeding and abnormal coagulation laboratory results, we administered the recombinant activated factor VII (rFVIIa), which was not approved for use in the treatment of post-CPB coagulopathy. After administration of rFVIIa, his coagulation data dramatically improved, the bleeding decreased, and the operation was able to be finished.

## Background

There have been increasing numbers of reports of “off-label” use of the recombinant activated factor VII (rFVIIa) to achieve hemostasis in various situations including traumatic intractable bleeding [1, 2], cardiac surgery [3, 4], and obstetrical massive bleeding [4]. However, there are few data regarding the appropriate use of rFVIIa, especially in pediatric patients. Here, we report a case of successful administration of rFVIIa for refractory post-cardiopulmonary bypass (CPB) bleeding in a child undergoing repeated cardiac surgery.

## Case presentation

A 2-year-and-7-month-old boy with 22q11.2 deletion syndrome (height, 86 cm; body weight, 10 kg) was scheduled for an emergent surgery to reconstruct the right ventricle-pulmonary artery (RV-PA) conduit to remove its compression on his right coronary artery, causing the right ventricular failure. He was associated with tetralogy of Fallot, pulmonary atresia, major aortopulmonary collateral arteries, and right aortic arch. He underwent a systemic-pulmonary shunt (left subclavian artery to central pulmonary artery) at 1 month of age and right unifocalization and right Blalock-Taussig shunt at 1 year and 3 months of age. When he was 2 years and 7 months old, he underwent RV-PA conduit and pulmonary artery plasty. After the procedure, because of the poor contraction of the right ventricle, weaning from CPB failed and extracorporeal membrane oxygenation (ECMO) was instituted, from which he was weaned on postoperative day 11.

Twenty-two days after surgery, cardiac catheterization revealed that his right coronary artery was compressed by the RV-PA conduit, and the emergent reconstitution of the RV-PA conduit was scheduled on the same day.

Anesthesia was induced with 20 μg of fentanyl and 10 mg of rocuronium, and repositioning of the RV-PA conduit was performed under CPB. He was successfully weaned from CPB with the support of inhaled nitric oxide (20 ppm), adrenaline (200 ng/kg/min), dopamine (10 μg/kg/min), and dobutamine (10 μg/kg/min). After reversal of heparin using protamine, persistent hemorrhage over 40 ml/kg/h continued for 3 h despite continuous massive administration of red blood cell (RBC), fresh frozen plasma (FFP), and platelets. At that time, abnormal coagulation tests were noted with prothrombin time (PT) of 36 % and international normalized ratio of PT (PT-INR) of 1.73, activated partial thromboplastin time (APTT) of 173 s, and fibrinogen was 172 mg/dl. The platelet count was 97,000/μL. As a rescue treatment to reduce bleeding and to correct his coagulation abnormality, a single dose of 100 μg/kg of rFVIIa was intravenously administered. One hour later, coagulation tests were almost normalized as shown in Table 1 and the hemorrhage decreased to 5–10 ml/kg/h, and the operation was finished with the open sternum. He was transferred to the Pediatric Intensive Care Unit (PICU).

**Table 1:** Coagulation profile before and after administration of rFVIIa

		Normal value	Before surgery	Prior to rFVIIa	1 h after rFVIIa	5 h after rFVIIa
Platelet	/μl	130–400 × 10^3^	438,000	97,000	110,000	218,000
PT	%	80–120	53	36	>120	81
PT-INR		0.9–1.1	1.37	1.73	0.82	1.10
APTT	s	25–45	64	173	44	32
Fibrinogen	mg/dl	180–360	308	172	229	228

*rFVIIa* recombinant activated coagulation factor VII, *PT* prothrombin time, *PT-INR* international normalized ratio of PT, *APTT* activated partial thromboplastin time

CPB time was 340 min, and his body temperature during CPB was 32 °C. Operation time was 639 min, and anesthesia time was 688 min. Total blood loss was 1480 ml, and required blood products were RBC 690, FFP 725, and platelets 250 ml, respectively.

Five hours after the administration of rFVIIa, coagulation data were comparable to the preoperative values (Table 1) and the total bleeding dose was less than 1 ml/kg/h. No additional rFVIIa was necessary. No thromboembolic events were noted after its administration.

Fifty-two days after the second surgery, his sternum and skin were able to be closed, and he was discharged from PICU on day 157.

### Discussion

We reported the use of rFVIIa in a child complicated with persistent bleeding after cardiac surgery under CPB. Because massive transfusion of FFP and platelets failed to control post-CPB bleeding, we decided to use rFVIIa though we knew that post-CPB coagulopathy was not a licensed indication. In this case, rFVIIa was administrated without previous parental informed consent for its use. The administration of rFVIIa dramatically improved his coagulation data and reduced blood loss.

Recombinant FVIIa is a hemostatic agent developed for the treatment of hemophilia A or B with inhibitors (antibodies) to coagulation factor VIII or IX. Currently, it is also approved to patients with congenital factor VII deficiency or the patients who have acquired antibodies to factor VIII, which is called as acquired hemophilia A. Furthermore, there have been increasing numbers of reports describing the off-label use of rFVIIa, which was effective in controlling refractory bleeding in nonhemophilic patients [1–4].

**Fig. 1:**
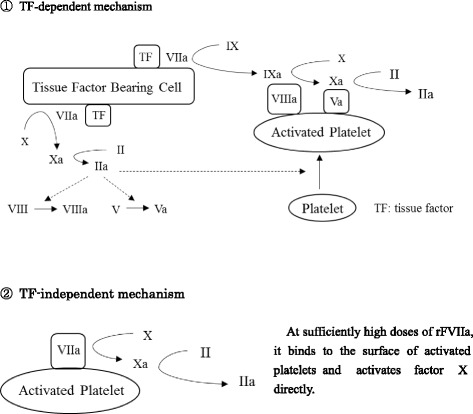
Mechanism of action of rFVIIa

Recombinant FVIIa is considered to work by two mechanisms (Fig. 1): tissue factor (TF)-dependent and independent mechanisms. In the TF-dependent mechanism, FVIIa/TF complexes activate coagulation factors IX and X and activated factor X combines with factor V, resulting in an increase of thrombin generation. In the TF-independent mechanism, FVIIa can directly activate factor X on the platelet surface without the presence of TF. These mechanisms activate and aggregate platelets, resulting in formation of stabilized clots [5].

Uncontrolled bleeding after CPB is a critical problem especially in young children undergoing complex cardiac repair. The proposed mechanisms of the post-CPB coagulopathy in these young patients include (1) large circuit priming volume compared to the circulating blood volume, leading to dilution of platelets and coagulation factors; (2) long, complex cardiac repairs with extensive suture lines; (3) effects of hypothermia on both plasma coagulation factors and platelet function; (4) consumption of coagulation factors due to tissue injury, contact activation, and transfusion of suctioned pericardial blood; and (5) residual heparin or other anticoagulants [3, 5]. Adding to the repeated procedures, our patient was on the ECMO until 11 days before and underwent cardiac catheterization a few hours before the surgery in the unstable hemodynamic and debilitating state. These conditions might have further influenced the coagulation derangement, as partly shown by the abnormal data of PT, PT-INR, and APTT before surgery (Table 1).

There is no consensus on how to manage post-CPB coagulopathy in pediatric patients. The transfusion of platelets is the initial treatment for ongoing bleeding after adequate heparin neutralization in many institutions [3, 6]. However, if bleeding continues despite platelet administration, institutions vary in their transfusion approach.

The congenital Cardiac Anesthesia Society (CCAS) Task Force recommends that rFVIIa can be considered for the management of post-CPB bleeding refractory to maximal standard hemostatic therapy in pediatric cardiac surgery [3]. This criterion for use of rFVIIa could be applied to our case.

It is not clear how the administration of rFVIIa worked dramatically in reducing bleeding in this case. The high dose of FVIIa might have stimulated the coagulation cascade by the activation of factors IX and X in combination with factors VIII and V through TF-dependent mechanism. Or the high dose of FVIIa might have stimulated the coagulation cascade, leading to subsequent thrombin generation through TF-independent mechanism. The latter mechanism seems more plausible because this pathway is enabled only when the high dose of FVIIa is provided and does not require the presence of factors V, VIII, and IX.

To exclude the possibility of acquired hemophilia A, the measurement of antibodies to coagulation factor VIII is required, which we did not perform in this case.

Concerning the dosage of rFVIIa, Pychyńska-Pokorska et al. reported that the effective dose in cardiac surgery was 131.7 ± 69.8 μg/kg in neonates, 104.6 ± 36.0 μg/kg in infants, and 44.6 ± 15.3 μg/kg in children older than 1 year of age [7]. A systematic literature review showed that the effective dose was 93.2 μg/kg, the frequency of administration was 2, and the most common administration interval was 2 h [3]. Since neonates and infants have immature coagulation system than adults [3], and the plasma clearance of rFVIIa is significantly higher in children than in adults (67 ml/kg/h in children, 36.6 ml/kg/h in adults) [3], children might require larger dose of rFVIIa than adults to achieve appropriate hemostasis. Considering the age and body weight of our patient, our dose of 100 μg/kg is comparable with the recommended dose.

One of the complications of administering rFVIIa is a thromboembolic event [8], especially in the patients with ECMO [3]. It is stated that we should properly assess the risk-benefit of administration rFVIIa under ECMO [9]. A thromboembolic event was not noted in our patient.

## Conclusions

We experienced a case of a successful “off-label” use of rFVIIa in a pediatric cardiac surgery. In the case of massive post-CPB bleeding refractory to standard hemostatic therapy, the administration of rFVIIa may be taken into consideration. To accumulate the successful cases is important to define the criteria of uses of rFVIIa in the future.

## Consent

Written informed consent was obtained from the parent of the patient for publication of this case report. A copy of the written consent is available for review by the Editor of this journal.
